# Diagnosis and management of metabolic acidosis: guidelines from a French expert panel

**DOI:** 10.1186/s13613-019-0563-2

**Published:** 2019-08-15

**Authors:** Boris Jung, Mikaël Martinez, Yann-Erick Claessens, Michaël Darmon, Kada Klouche, Alexandre Lautrette, Jacques Levraut, Eric Maury, Mathieu Oberlin, Nicolas Terzi, Damien Viglino, Youri Yordanov, Pierre-Géraud Claret, Naïke Bigé, for the Société de Réanimation de Langue Française (SRLF), the Société Française de Médecine d’Urgence (SFMU)

**Affiliations:** 1 Département de Médecine Intensive et Réanimation, CHU Montpellier, 34000 Montpellier, France; 2 INSERM U-1046, CNRS U-9234 (PhyMedExp), Université de Montpellier, Montpellier, France; 3 Pôle Urgence, CH du Forez, 42605 Montbrison, France; 4 Réseau d’urgence Ligérien Ardèche Nord (REULIAN), Centre Hospitalier Le Corbusier, 42700 Firminy, France; 5 Département de Médecine d’urgence, Centre Hospitalier Princesse-Grace, Avenue Pasteur, 98012 Monaco, France; 6 Unité de Médecine Intensive et Réanimation, Hôpital Universitaire Saint-Louis, Assistance Publique–Hôpitaux de Paris, Avenue Claude-Vellefaux, 75010 Paris, France; 7 Faculté de Médecine, Université Paris-Diderot, Sorbonne–Paris-Cité, Paris, France; 8 France Inserm, ECSTRA Team, UMR 1153, Centre d’Epidémiologie et de Biostatistique, CRESS, Biostatistics and Clinical Epidemiology, Sorbonne–Paris-Cité, Paris, France; 9 Département de Médecine Intensive–Réanimation, CHU Lapeyronie, 371, Avenue Doyen-Gaston-Giraud, 34295 Montpellier, France; 10 Réanimation, Centre Jean-Perrin, CHU de Clermont-Ferrand, 63000 Clermont-Ferrand, France; 11 LMGE, UMR CNRS 6023, Université Clermont-Auvergne, Clermont-Ferrand, France; 12 Département de Médecine d’urgence, CHU de Nice, Hôpital Pasteur-II, 30, Avenue de la Voie Romaine, 06000 Nice, France; 13 UFR de Médecine, Université de Nice Côte d’Azur, Avenue de Vallombrose, 06000 Nice, France; 14 Service de Médecine Intensive–Réanimation, Hôpital Saint-Antoine, Assistance Publique–Hôpitaux de Paris, 184, Rue du Faubourg-Saint-Antoine, 75571 Paris Cedex 12 Paris, France; 15 Sorbonne Université, Université Pierre-et-Marie Curie-Paris-VI, Paris, France; 16 Inserm U1136, 75012 Paris, France; 17 Structure des Urgences, Centre Hospitalier de Cahors, 335, Rue Wilson, 46000 Cahors, France; 18 Service de Médecine Intensive–Réanimation, Centre Hospitalier Universitaire de Grenoble, Université de Grenoble, Grenoble, France; 19 Inserm, U1042, Université Grenoble-Alpes, HP2, 38000 Grenoble, France; 20 Service des Urgences Adultes, CS 10217, CHU Grenoble-Alpes, 38043 Grenoble Cedex 09 Grenoble, France; 21 Inserm U1042, Laboratoire HP2 Hypoxie-Physiopathologies, Université Grenoble-Alpes, Grenoble, France; 22 Faculté de Médecine, Sorbonne Universités, 75013 Paris, France; 23 Inserm, U1153, Université Paris-Descartes, 75006 Paris, France; 24 Service des Urgences, Hôpital Saint-Antoine, Assistance Publique–Hôpitaux de Paris (AP–HP), 75012 Paris, France; 25 Pôle Anesthésie Réanimation Douleur Urgences, Centre Hospitalier Universitaire de Nîmes, 4, Rue du Professeur-Robert-Debré, 30029 Nîmes, France

**Keywords:** Metabolic acidosis, Blood gas analysis, Anion gap, Hyperlactatemia, Ketoacidosis, Sodium bicarbonate, Renal replacement therapy

## Abstract

Metabolic acidosis is a disorder frequently encountered in emergency medicine and intensive care medicine. As literature has been enriched with new data concerning the management of metabolic acidosis, the French Intensive Care Society (Société de Réanimation de Langue Française [SRLF]) and the French Emergency Medicine Society (Société Française de Médecine d’Urgence [SFMU]) have developed formalized recommendations from experts using the GRADE methodology. The fields of diagnostic strategy, patient assessment, and referral and therapeutic management were addressed and 29 recommendations were made: 4 recommendations were strong (Grade 1), 10 were weak (Grade 2), and 15 were experts’ opinions. A strong agreement from voting participants was obtained for all recommendations. The application of Henderson–Hasselbalch and Stewart methods for the diagnosis of the metabolic acidosis mechanism is discussed and a diagnostic algorithm is proposed. The use of ketosis and venous and capillary lactatemia is also treated. The value of pH, lactatemia, and its kinetics for the referral of patients in pre-hospital and emergency departments is considered. Finally, the modalities of insulin therapy during diabetic ketoacidosis, the indications for sodium bicarbonate infusion and extra-renal purification as well as the modalities of mechanical ventilation during severe metabolic acidosis are addressed in therapeutic management.

## Introduction

The Henderson–Hasselbalch method defines metabolic acidosis by the presence of an acid–base imbalance associated with a plasma bicarbonate concentration below 20 mmol/L. The association of this imbalance with decreased pH is called “acidemia,” which is often described as severe when the pH is equal to or below 7.20.

Metabolic acidosis is a frequent event in patients receiving emergency treatment or intensive care. Physicians have at their disposal numerous plasma and urine tests to characterize metabolic acidosis, determine its etiology, and refer patients. Acute metabolic acidosis may accompany various diseases and be associated with organ failure, in particular respiratory (increased ventilatory demand) and cardiovascular (arterial vasodilation, decreases in cardiac inotropism and cardiac output, ventricular arrythmia) [1–3]. The role of acute metabolic acidosis in these organ failures is mostly suggested by experimental studies in animals or in vitro, as few clinical studies in humans are available [1].

The last consensus conference on the “correction of metabolic acidosis in intensive care” was published in 1999 by the Société de Réanimation de Langue Française (SRLF), with the participation of the Société Française d’Anesthésie et de Réanimation (SFAR), the Société Francophone d’Urgences Médicales (SFUM), the Groupe Francophone de Réanimation et Urgences Pédiatriques (GFRUP), Samu de France, the Société Française de Nutrition Entérale et Parentérale, and the Société de Néphrologie. Point-of-care testing has since grown and enables clinicians to access blood gas measurements very quickly, including in pre-hospital settings. In addition, new data on diagnosis and prognostic tools and the treatment of metabolic acidosis have enriched literature. This is why the SRLF and the Société Française de Médecine d’Urgence (SFMU) propose these formal guidelines on the diagnosis and management of metabolic acidosis. Through analysis of the level of evidence in the literature, the purpose of these guidelines is to specify the diagnostic strategy, referral of patients, and therapeutic management in pre-hospital settings, in the emergency room, and in intensive care.

## Method

The guidelines were drawn up by a group of twelve experts convened by the SRLF and the SFMU. The group’s agenda was defined beforehand. The organizing committee first defined the questions to be addressed with the coordinators and then designated the experts in charge of each question. The questions were formulated according to a Patient Intervention Comparison Outcome (PICO) format after a first meeting of the expert group. Literature was analyzed and the guidelines were formulated using Grade of Recommendation Assessment, Development and Evaluation (GRADE) methodology. A level of evidence was defined for each bibliographic reference cited as a function of the type of study and could be reassessed in light of the methodological quality of the study. An overall level of evidence was determined for each endpoint, taking into account the level of evidence of each bibliographic reference, the between-study consistency of the results, the direct or indirect nature of the results, and cost analysis. Three levels of proof were used (Table 1):

**Table 1:** Recommendations according to the GRADE methodology

	Recommendation according to the GRADE methodology	
High level of evidence	Strong recommendation “… should be done…”	Grade 1+
Moderate level of evidence	Optional recommendation “… should probably be done…”	Grade 2+
Insufficient level of evidence	Recommendation in the form of an expert opinion “The experts suggest…”	Expert opinion
Moderate level of evidence	Optional recommendation “… should probably not be done…”	Grade 2−
High level of evidence	Strong recommendation “… should not be done…”	Grade 1−

- A high overall level of evidence enabled formulation of a “strong” recommendation (should be done… GRADE 1+, should not be done… GRADE 1−).
- A moderate, low, or very low overall level of evidence led to the drawing up of an “optional” recommendation (should probably be done… GRADE 2+, should probably not be done… GRADE 2−).
- When literature was inexistent or insufficient, the question could be the subject of a recommendation in the form of an expert opinion (the experts suggest…).

The proposed recommendations were presented and discussed one by one. The aim was not necessary to reach a single and convergent opinion of the experts on all the proposals, but to define the points of agreement and the points of disagreement or uncertainty. Each expert then reviewed and rated each recommendation using a scale of 1 (complete disagreement) to 9 (complete agreement). The collective rating was done using a GRADE grid. To approve a recommendation regarding a criterion, at least 50% of the experts had to be in agreement and less than 20% in disagreement. For an agreement to be strong, at least 70% of the experts had to be in agreement. In the absence of strong agreement, the recommendations were reformulated and rated again, with a view to reaching a consensus. Only expert opinions that elicited strong agreement were kept.

## Areas of recommendations

Three areas were defined: diagnostic strategy, referral of patients, and therapeutic management. A bibliographic search was conducted using the MEDLINE database via PubMed and the Cochrane database. For inclusion in the analysis, the publications had to be written in English or French. The analysis focused on all literature data without imposing a date limit, according to an order of appraisal ranging from meta-analyses to randomized trials to observational studies. The size of the study populations and the relevance of the research were considered for each study.

### Summary of results

The summary of the results by the experts according to the GRADE method led to the drawing up of 29 guidelines. Of these guidelines, 4 had a high level of evidence (GRADE 1±) and 10 a low level of evidence (GRADE 2±). The GRADE method was inapplicable to 15 guidelines, which resulted in expert opinions. After two rounds of scoring, a strong agreement was reached for all guidelines. Table 2 provides a summary of the recommendations.

**Table 2:** Summary of recommendations

	Recommendation	Level of evidence
Diagnostic strategy		
R1.1	The experts suggest that arterial blood gas measurements be performed in patients with a decreased plasma bicarbonate level so as to eliminate respiratory alkalosis, confirm the diagnosis of metabolic acidosis, and test for mixed acidosis	Expert opinion
R1.2	Measurement of base deficit should probably not be preferred to that of plasma bicarbonate in identifying patients at risk of metabolic acidosis	Grade 2−
R1.3	The anion gap corrected for albumin should probably be used rather than the uncorrected anion gap to differentiate acidosis related to acid load from acidosis related to base deficit	Grade 2+
R1.4	The experts suggest first applying the Henderson–Hasselbalch method using the plasma anion gap corrected for albumin for the diagnosis of the mechanism of metabolic acidosis. However, the Stewart method gives insight into situations unexplained by the Henderson–Hasselbalch method: acid–base imbalance secondary to blood sodium and chloride imbalance and complex disorders	Expert opinion
R1.5	The experts suggest using an algorithm to improve the etiological diagnosis of metabolic acidosis	Expert opinion
R1.6	The experts suggest that the urinary anion gap should only be calculated in metabolic acidosis without unmeasured anions or obvious etiology	Expert opinion
R1.7	The experts suggest that measurement of urinary pH should be restricted to patients with metabolic acidosis without unmeasured anions or obvious etiology, and with a strong clinical suspicion of tubular acidosis	Expert opinion
R1.8	The experts suggest that a normal value of venous lactate discounts hyperlactatemia	Expert opinion
R1.9	Arterial lactate should probably be measured to confirm hyperlactatemia in case of increased venous lactate	Grade 2+
R1.10	Capillary blood lactate should not be measured to diagnose hyperlactatemia	Grade 1−
R1.11	Capillary blood ketones rather than urine ketones should be measured when diagnosing ketoacidosis	Grade 1+
Patient assessment and referral		
R2.1	The pH value should probably not be used alone to identify critically ill patients	Grade 2−
R2.2	Hyperlactatemia, whatever its value, should be considered as a marker of severity in initial treatment. Diagnostic and therapeutic management should be rapid and multidisciplinary if needed	Grade 1+
R2.3	Increase in blood lactate should probably be controlled in the first hours of management so as to assess the response to treatment	Grade 2+
R2.4	The experts suggest close monitoring of patients with diabetic ketoacidosis, ideally in an Intensive Care Unit	Expert opinion
Therapeutic management		
R3.1	Insulin should probably be administered intravenously rather than subcutaneously in patients with diabetic ketoacidosis	Grade 2+
R3.2	An insulin bolus should probably not be administered before starting continuous intravenous insulin therapy in patients with diabetic ketoacidosis	Grade 2−
R3.3	Low continuous intravenous insulin doses should probably be administered in the treatment of diabetic ketoacidosis	Grade 2+
R3.4	The experts suggest using an initial dosage of 0.1 IU/kg/h without exceeding 10 IU/h, and to increase it in the absence of hypokalemia, if the targets for correction of blood ketones (0.5 mmol/L/h), bicarbonate (3 mmol/L/h), and capillary blood glucose (3 mmol/L/h) are not reached after the first hours of treatment	Expert opinion
R3.5	The experts suggest administering sodium bicarbonate to compensate for gastrointestinal or renal base loss in case of poor clinical tolerance	Expert opinion
R3.6	Sodium bicarbonate should probably be administered to intensive care patients with severe metabolic acidemia (pH ≤ 7.20, PaCO_2_ < 45 mmHg) and moderate to severe acute renal insufficiency, so as to improve prognosis	Grade 2+
R3.7	Sodium bicarbonate should not be administered routinely in the therapeutic management of circulatory arrest, apart from pre-existing hyperkalemia or poisoning by membrane stabilizers	Grade 1−
R3.8	Sodium bicarbonate should probably not be administered to patients with diabetic ketoacidosis	Grade 2−
R3.9	The experts suggest administering sodium bicarbonate in the therapeutic management of salicylate poisoning, whatever the pH value	Expert opinion
R3.10	In case of shock and/or acute renal insufficiency, the experts suggest initiation of renal replacement therapy if the pH is below or equal to 7.15 in the absence of severe respiratory acidosis and despite appropriate treatment	Expert opinion
R3.11	In case of lactic acidosis suggestive of metformin poisoning, the experts suggest early initiation of renal replacement therapy when there is organ dysfunction or in the absence of improvement in the first hours of therapeutic management	Expert opinion
R3.12	In case of methanol or ethylene glycol poisoning, the experts suggest initiation of renal replacement therapy if the anion gap is above 20 mEq/L or if there is renal insufficiency or visual impairment	Expert opinion
R3.13	In metabolic acidosis associated with salicylic acid poisoning, the experts suggest initiation of renal replacement therapy when there is neurological involvement and/or if the salicylic acid concentration is above 6.5 mmol/L (90 mg/dL) and/or if the pH is less than or equal to 7.20	Expert opinion
R3.14	The experts suggest compensating for acidemia by increasing respiratory frequency without inducing intrinsic positive end-expiratory pressure, with a maximum of 35 cycles/min and/or a tidal volume up to 8 mL/kg of body mass, and by monitoring plateau pressure. The aim of ventilation is not to normalize pH. A target pH greater than or equal to 7.15 seems reasonable. Medical treatment of metabolic acidosis and of its cause should be envisaged concomitantly, as ventilatory compensation can only be symptomatic and temporary	Expert opinion

### First area: Diagnostic strategy

#### Should arterial blood gas measurements be performed in the patients with a decreased plasma bicarbonate level when diagnosing acid–base imbalance?

R1.1—The experts suggest that arterial blood gas measurements be performed in patients with a decreased plasma bicarbonate level so as to eliminate respiratory alkalosis, confirm the diagnosis of metabolic acidosis, and test for mixed acidosis (EXPERT OPINION).

*Rationale* Acidosis is a pathophysiological process that may account for a decrease in blood pH that defines acidemia. Two main mechanisms may be responsible: a decrease in plasma bicarbonate, defining metabolic acidosis, and an increase in PaCO_2_, defining respiratory acidosis. In the case of metabolic acidosis, the decrease in plasma bicarbonate either reflects the intervention of the buffer system related to an accumulation of non-respiratory acids, or excessive loss of bicarbonate.

The pH can be kept normal through the decrease in PaCO_2_ obtained by compensating hyperventilation. Acidemia occurs when respiratory compensation is insufficient. The PaCO_2_ value that maintains a normal pH, called the expected PaCO_2_, can be calculated using the formula: expected PaCO_2_ = 1.5 × [HCO_3_^−^] + 8 ± 2 mmHg [4, 5]. Blood gas measurements can be used to assess respiratory compensation and so detect mixed acidemia: pH < 7.38, HCO_3_^−^ < 20 mmol/L and measured PaCO_2_ > expected PaCO_2_.

As the decrease in plasma bicarbonate may also be related to a compensation mechanism of respiratory alkalosis [6], blood gas measurements would allow elimination of respiratory alkalemia: pH > 7.42 and PaCO_2_ < 38 mmHg.

Most studies that have measured the agreement and limits of agreement between venous and arterial blood gas measurements did not evaluate the clinical superiority of one method with respect to another for diagnosis of metabolic acidosis and were conducted on moderate-sized groups of selected patients. A meta-analysis of studies comparing arterial and venous blood gas measurements in patients in emergency rooms found excellent agreement between the arterial and venous pH (mean difference − 0.033 [95% CI − 0.039 to 0.027]) [7]. A single study of the management of ketoacidosis in the emergency room found that arterial blood gas measurements altered treatment in 3.7% of cases and changed disposition in 1% of cases [8]. These modifications were deemed negligible and the authors concluded that the techniques were equivalent. Very good agreement between arterial and venous measurements of base deficit was also found in trauma patients [9, 10]. Similar results, with a mean pH difference of 0.03 [95% CI − 0.02 to 0.08], were found in critically ill patients with metabolic acidosis of various causes, except ketoacidosis [11].

However, the agreement between venous and arterial blood gas measurements was much poorer for PaCO_2_. In a meta-analysis of studies comparing arterial and venous PaCO_2_ values in patients in the emergency room, the mean difference was 4.41 mmHg [95% CI 2.55–6.27], with limits of agreement ranging from − 20.4 to 25.8 mmHg [7].

### Is base deficit a better measurement than plasma bicarbonate in diagnosing metabolic acidosis?

R1.2—Measurement of base deficit should probably not be preferred to that of plasma bicarbonate in identifying patients at risk of metabolic acidosis (GRADE 2−, STRONG AGREEMENT).

*Rationale* Clinical data are scarce and limited (observational, retrospective studies) [12–14]. The two largest studies show that if the control group is of patients with a base excess (BE) of −5 mmol/L, corresponding to a base deficit of 5 mmol/L, plasma bicarbonate below 20 mmol/L is a good diagnostic indicator of metabolic acidosis [13, 14]. BE corresponds to the quantity of strong acid (or of strong base in the case of a metabolic acidosis) that should be added in vitro to 1 L of plasma to normalize the pH to 7.40, with a PaCO_2_ of 40 mmHg and a temperature of 37 °C. There are several methods of calculating BE, but they all use plasma bicarbonate as the main component. Standard base excess (SBE) calculated using the van Slyke equation* takes into account a hemoglobin concentration of 5 g/dL which is the theoretical hemoglobin concentration in the extracellular space of bicarbonate distribution. The van Slyke equation for SBE is the most used clinically, but is not used in comparative studies with plasma bicarbonate. As BE is always calculated from plasma bicarbonate, the correlation between plasma bicarbonate and BE (and hence base deficit) is very strong.

* Van Slyke equation:

Base excess = (HCO_3_^−^–24.4) + (2.3 × Hb + 7.7) × (pH − 7.4) × (1 − 0.023 × Hb), with Hb in g/dL.

### In case of metabolic acidosis, is the plasma anion gap corrected for albumin better than the uncorrected plasma anion gap in differentiating acid excess from base deficit?

R1.3—The anion gap corrected for albumin should probably be used rather than the uncorrected anion gap to differentiate acidosis related to acid load from acidosis related to base deficit (GRADE 2+, STRONG AGREEMENT).

*Rationale* Although most clinical data are prospective, they are scarce and observational. Comparisons between the corrected anion gap* (cAG) and the uncorrected anion gap** (AG) show either no difference [15, 16] or superiority of cAG [17–19]. Most authors consider that the pathological threshold is cAG or AG > 12 mmol/L. The physiological AG is mainly composed of phosphate and albuminate (weak anion from blood albumin). Consequently, hypoalbuminemia leads to a decrease in plasma albuminate and so to a decrease in AG. Hence, a normal AG associated with hypoalbuminemia corresponds to the presence of plasma acids, which replace albuminate to normalize AG. Taking the albumin level into account in the calculation of AG unmasks plasma acids when there is hypoalbuminemia. So, cAG is greater than AG, particularly in a population of patients with a high risk of hypoalbuminemia, as is the case for patients in intensive care or patients with malnutrition, hepatopathy, chronic inflammation, or urinary loss of albumin.

* cAG = AG + (40 − [albuminemia]) × 0.25, with albuminemia in g/L.

** AG = Na^+^ − (Cl^−^ + HCO_3_^−^) = 12 ± 4 mmol/L (or AG = (Na^+^ + K^+^) − (Cl^−^ + HCO_3_^−^) = 16 ± 4 mmol/L).

### Is the Stewart method equivalent to the Henderson–Hasselbalch method using the anion gap corrected for albumin for the diagnosis of the mechanism of metabolic acidosis?

R1.4—The experts suggest first applying the Henderson–Hasselbalch method using the anion gap corrected for albumin for the diagnosis of the mechanism of metabolic acidosis. However, the Stewart method provides insight into situations unexplained by the Henderson–Hasselbalch method: acid–base imbalance secondary to blood sodium and chloride imbalance and complex disorders (EXPERT OPINION).

*Rationale* The Henderson–Hasselbalch approach using the anion gap corrected for albumin and the Stewart method was proposed for the identification of causes of acid–base imbalances [17, 20–24]. The anion gap (AG) (or unmeasured anions) requires just a simple and rapid calculation*. At equilibrium, AG does not account for low-level cations that are not measured routinely (Mg^2+^, Ca^2+^, H^+^) and is essentially explained by anions not determined by blood electrolyte measurements (essentially albuminate and phosphate). An increase in AG classically indicates the accumulation of an acid whose anion is not chloride and theoretically corresponds to the accumulation of one of the following compounds: lactate, acetoacetate, hydroxybutyrate, oxalate, glycolate, formate, salicylate, sulfate. However, this reasoning implies that the value of unmeasured anions, essentially albuminate, and more rarely phosphate (Pi), is normal. Indeed, hypoalbuminemia results in a decrease in unmeasured anions and will reduce the anion gap [25]. The accumulation of an anion like lactate or acetoacetate may therefore be missed, because AG is falsely normal. Albumin-correction AG (cAG)** identifies most situations where there is accumulation of an anion other than chloride and hypoalbuminemia [26].

The Stewart approach assumes that the acid–base balance is based on a dissociation of water molecules that depends on three independent variables: PaCO_2_, the strong ion difference which corresponds to the difference between strong cations and strong anions (apparent SID (appSID) = Na^+^ + K^+^ + Ca^2+^ + Mg^2+ ^− Cl^−^) and the sum of non-volatile weak acids present in dissociated or undissociated form (Atot) defined by [albumin × (0.123 × pH − 0.631) + [Pi × (0.309 × pH − 0.469]. Use is made of the effective SID: eff SID = HCO_3_^−^ + albuminate^−^ + Pi^−^ = HCO_3_^−^ + Atot. Respiratory acid–base disturbances are exclusively defined by increased PaCO_2_. The approach to metabolic acid–base disturbances requires calculation of the strong ion gap (SIG), which is equal to appSID—eff SID. In the Stewart model, given that electrical neutrality must be respected, the variation in bicarbonate concentration is the consequence of the acid–base disturbance and not its cause unlike the hypothesis of Henderson–Hasselbalch model. A positive SIG indicates the presence of unmeasured anions and so of metabolic acidosis [27–29]. The Stewart approach appears at least equivalent to the Henderson–Hasselbalch approach in the case of accumulation of endogenous or exogenous acid or loss of bicarbonate [17, 20, 22, 25]. However, the Stewart approach sheds light on metabolic disorders secondary to blood sodium and chloride levels, such as hyperchloremic acidosis associated with saline fluid resuscitation, which the Henderson–Hasselbalch approach explains less easily [23, 26], and on complex disorders (hyperlactatemia at normal pH and BE) [30–32].

* AG = Na^+^ − (Cl^−^ + HCO_3_^−^) = 12 ± 4 mmol/L (or AG = (Na^+^ + K^+^) − (Cl^−^ + HCO_3_^−^) = 16 ± 4 mmol/L).

** cAG = AG + (40 − [blood albumin]) × 0.25, with blood albumin in g/L.

### Does the use of a diagnostic algorithm improve etiological diagnosis of metabolic acidosis?

**Fig. 1:**
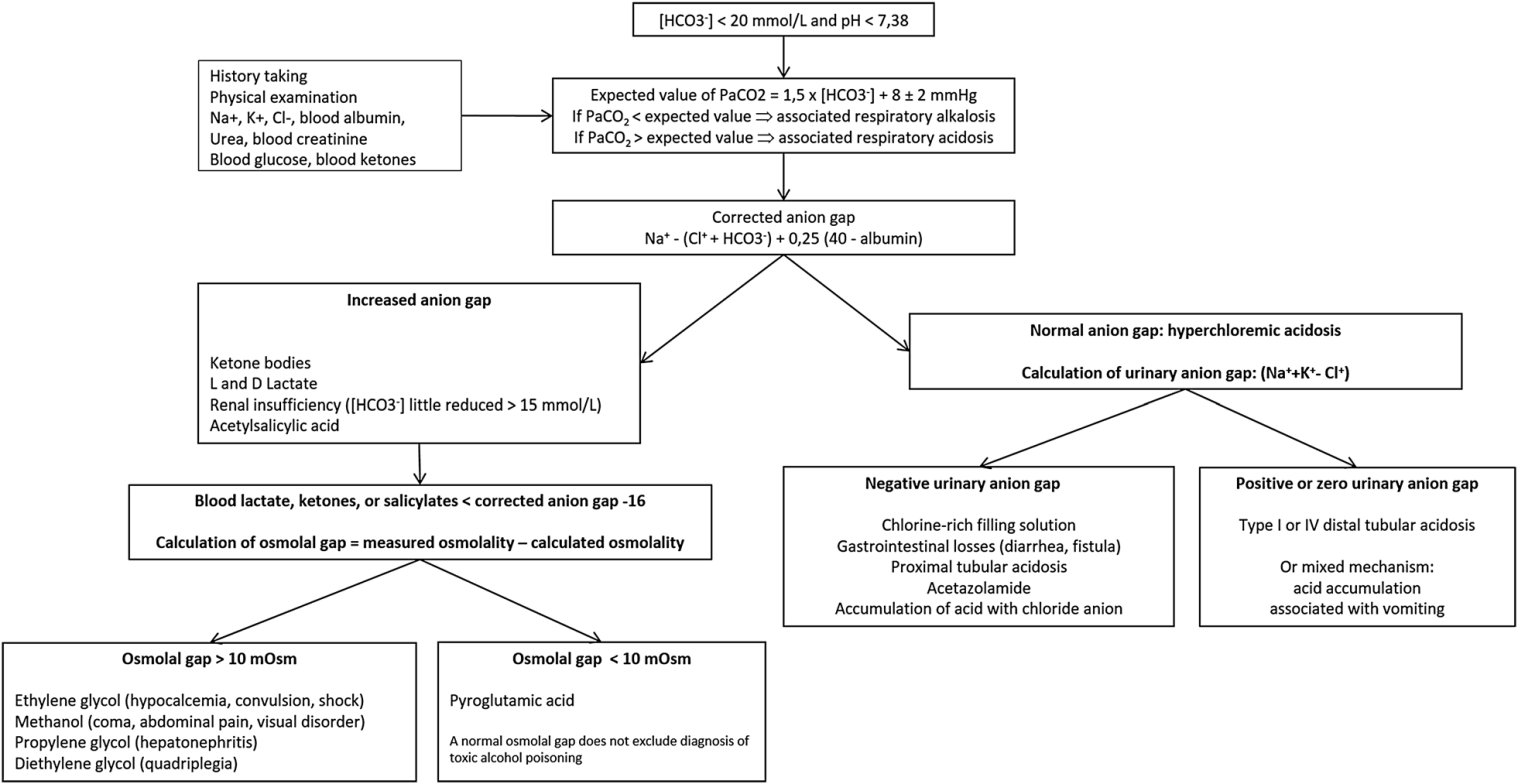
Algorithm recommended by the experts for etiological diagnosis of metabolic acidemia (EXPERT OPINION)

R1.5—The experts suggest using an algorithm to improve the etiological diagnosis of metabolic acidosis (EXPERT OPINION) (Fig. 1).

*Rationale* Few studies have evaluated the diagnostic impact of the use of an algorithm in metabolic acidosis, so it is difficult to provide a well-argued answer to the question. It is always essential to collect data by clinical history taking and physical examination [33]. Apart from the exceptions mentioned below, the use of an algorithm could subsequently be used to investigate simple settings of acidosis [28]. However, it is important to know if there are artifacts [34] or atypical [35], complex [33], or misleading clinical pictures [30, 36]. Acetylsalicylic acid poisoning associates initial respiratory alkalosis with metabolic acidosis responsible for an increase in the anion gap which is only partly explained by the accumulation of acetylsalicylic acid [33]. The increased anion gap acidosis seen in ethylene glycol poisoning is in part due to the accumulation of glycolic acid, which some laboratory analyzers misidentify as lactate [34, 37]. Diabetic ketoacidosis may be accompanied by hyperchloremic acidosis at hospital admission or a few hours after admission [35]. The association of lactate production with vomiting may lead to the clinical picture of metabolic alkalosis [30]. Abundant infusion of chloride-rich fluid during circulatory insufficiency associated with hyperlactatemia produces hyperchloremic acidosis [30, 38]. It is important to remember that the respiratory compensation observed in acute metabolic acidosis cannot correct the pH value beyond 7.40 (Fig. 1). Main causes of hyperlactatemia are listed in Table 3.

**Table 3:** Main causes of hyperlactatemia suggested by the experts (EXPERT OPINION)

Type A Severe anemia Septic, hemorrhagic, cardiogenic shock CO poisoning Organ ischemia Convulsions Intense physical exercise
Type B Sub-type B1—Underlying primary diseases Cancer and hemopathy Decompensated diabetes HIV infection Liver failure Sepsis Severe malaria attack Sub-type B2—Medication and toxins Alcohol Beta-adrenergic agents Cyanide and cyanogenic compounds Diethyl ether Fluorouracil (5-FU) Halothane Iron Isoniazid Linezolid Metformin Nalidixic acid Niacin (vitamin B3 or nicotinic acid) Nucleoside reverse transcriptase inhibitors Paracetamol Propofol Psychostimulants: cocaine, amphetamines, cathinones Salicylates Strychnine Sugars: fructose, sorbitol, xylitol Sulfasalazine Total parenteral nutrition Valproic acid Vitamin deficiency: thiamine (vitamin B1) and biotin (vitamin B8) Sub-type B3—Inborn errors of metabolism Fructose-1,6-diphosphatase deficiency Glucose-6-phosphatase deficiency (von Gierke disease) Kearns–Sayre syndrome MELAS syndrome MERRF syndrome Methylmalonic acidemia (methylmalonyl-CoA mutase deficiency) Pearson syndrome Pyruvate carboxylase deficiency Pyruvate dehydrogenase deficiency

If no etiology is found, a hereditary metabolic disorder may be considered.

### Should the urinary anion gap always be calculated in metabolic acidosis?

R1.6—The experts suggest that the urinary anion gap should only be calculated in metabolic acidosis without unmeasured anions or obvious etiology (EXPERT OPINION).

*Rationale* The urinary anion gap, calculated as the sum of measured anions and cations (Na^+^ + K^+^ + Cl^−^), was proposed for estimation of the urinary excretion of ammonium (NH4^+^) [39–41]. In acidosis with a normal anion gap, ammonium distinguishes between acidosis related to gastrointestinal loss of bicarbonate (negative urinary anion gap), acidosis linked to tubular acidosis, or hyporeninemic hypoaldosteronism (zero or increased anion gap) [39–41].

The diagnostic utility of the urinary anion gap, notably in the emergency room or in intensive care is, however, questionable. First, outside intensive care, the performance of this index is only validated by studies of very low level of evidence [6, 40, 41]. The urinary anion gap seems to be correlated with NH4^+^ excretion [28], but the correlation is weak, the reported variability is substantial, and none of the available studies adjust for confounding factors or report the diagnostic performance [6, 40, 41]. Second, the discriminating character and the contribution to diagnosis are not evaluated. Lastly, most studies conducted in the emergency room or in intensive care have not evaluated this parameter [22, 23, 27].

A single study suggests a high prevalence of tubular acidosis in intensive care. It is of low level of evidence, as the parameter used (urinary anion gap) is also a diagnostic criterion of the expected clinical picture (tubular acidosis) [42].

### Should urinary pH always be measured in metabolic acidosis?

R1.7—The experts suggest that measurement of urinary pH should be restricted to patients with metabolic acidosis without unmeasured anions or obvious etiology, and with a strong clinical suspicion of tubular acidosis (EXPERT OPINION).

*Rationale* The measurement of urinary pH is less well validated than calculation of the urinary anion gap. Its diagnostic value is controversial and seems more restricted [28, 36, 39, 41].

### Is measurement of venous lactate less effective than measurement of arterial lactate in diagnosing hyperlactatemia?

R1.8—The experts suggest that a normal value of venous lactate discounts hyperlactatemia (EXPERT OPINION).

R1.9—Arterial lactate should probably be measured to confirm hyperlactatemia in case of increased venous lactate (GRADE 2+, STRONG AGREEMENT).

*Rationale* Measurement of arterial lactate is the reference method for determining blood lactate. Venous blood is more easily sampled than arterial blood and less painful for the patient. Several studies have evaluated the agreement between measurements of venous and arterial blood lactate. A 2014 meta-analysis included three of these studies [7]. They were prospective or retrospective cohort studies involving patient selection bias (non-consecutive patients), blood lactate was rarely above 4 mmol/L, and the measurement equipment and sampling conditions differed from one study to another. The mean bias in the results ranged from − 0.016 to 1.06 mmol/L. The Bland–Altman limits of agreement ranged from − 1.51 to 2.65 mmol/L. These biases and limits, reported for the usual lactate values, show that measurement of venous lactate is inadequate for the diagnosis of hyperlactatemia.

Measurement of venous lactate has also been assessed in prognostic cohort studies of patients with severe trauma, suspected septic shock, or admitted to the emergency room [43–46]. The study populations were not all comparable and the results were not unequivocal, in particular for venous lactate values below 4 mmol/L. On the other hand, it seems that a venous lactate value above 4 mmol/L was strongly associated with an increased risk of death.

In conclusion, whereas the measurement of venous lactate can be useful in determining the prognosis, the literature data do not support its use in the diagnosis of hyperlactatemia.

#### Is the measurement of capillary blood lactate as effective as measurement of arterial lactate in diagnosing hyperlactatemia?

R1.10—Capillary blood lactate should not be measured to diagnose hyperlactatemia (GRADE 1−, STRONG AGREEMENT).

*Rationale* The measurement of capillary blood lactate is less invasive and faster than measurement of arterial lactate. Several cohort studies have compared these two methods of measurement. Mean bias ranged from − 0.99 to 2.4 mmol/L. The Bland–Altman limits of agreement ranged from − 5.6 to 5.4 mmol/L [47–51]. These results are difficult to analyze because different measuring equipment was used and there are inconsistencies between the results [52]. Measurement of capillary blood lactate is therefore insufficiently efficient and does not allow sufficiently accurate determination of arterial lactate.

Measurement of capillary blood lactate has been proposed as a prognostic tool. Most studies, conducted before hospital admission or upon admission to the emergency room, have combined several sampling techniques (venous and capillary). Few studies have analyzed measurement of capillary blood lactate as a prognostic tool for patients with severe trauma or suspected septic shock [44, 47, 53]. These studies were of low level of evidence and do not allow a conclusion to be drawn regarding the prognostic value of measurement of capillary blood lactate. 

Hyperlactatemia occurs when there is an imbalance between lactate production and clearance [54]. Traditionally, the causes of hyperlactatemia have been divided into two groups: associated with tissue hypoxia (type A) and without tissue hypoxia (type B) [55, 56]. However, the mechanism can be mixed and the same etiology can be found in both groups [57].

#### Is measurement of capillary blood ketones more effective than measurement of urine ketones in diagnosing ketoacidosis?

R1.11—Capillary blood ketones rather than urine ketones should be measured when diagnosing ketoacidosis (GRADE 1+, STRONG AGREEMENT).

*Rationale* Studies comparing urine ketones and blood ketones are all observational. There is just one randomized controlled prospective study, but it evaluated the incidence of hospitalization/emergency assessment among patients with type 1 diabetes depending on self-measurement of blood ketones or urine ketones [58]. Most studies have included patients presenting to the emergency room because of a hyperglycemic episode (blood glucose generally > 2.5 g/L). The diagnostic criteria of diabetic ketoacidosis varied from one study to another, which makes comparison difficult. Whatever their quality, all the studies found greater specificity and a quicker diagnostic result with capillary blood ketones, for a comparable sensitivity. In addition, urine ketones may persist in the absence of significant blood ketones. Lastly, measurement of urine ketones determines only acetoacetate, whereas measurement of blood ketones determines solely beta-hydroxybutyrate, which is the predominant ketone body in the case of diabetic ketosis. Depending on the various cut-offs reported, blood ketones above 3 mmol/L associated with hyperglycemia constitute a good diagnostic criterion of diabetic ketoacidosis [59–63].

### Second area: Patient assessment and referral

#### In case of metabolic acidosis, is the pH value useful to identify critically ill patients?

R2.1—The pH value should probably not be used alone to identify critically ill patients (GRADE 2−, STRONG AGREEMENT).

*Rationale* The blood pH is a fundamental laboratory parameter. Its value depends not only on metabolic or respiratory variations, but also on the site of arterial, venous, or capillary blood sampling. Clinical studies of the prognostic utility of blood pH in emergency medicine have used analysis of venous or arterial blood. The emergence of new tools for point-of-care measurement of pH has yielded recent published studies of its prognostic value before hospital admission. These observational studies essentially related to non-traumatic cardiac arrest and most failed to show a prognostic utility of the isolated measurement of pH [64, 65]. However, there is a need to assess pH combined with other clinical and biochemical parameters. Most in-hospital studies were observational, were limited by small numbers of patients, and assessed very different diseases (cardiac arrest, trauma, pneumonia, diabetic ketoacidosis). Most of them failed to show any prognostic value of pH measurement [8, 66, 67]. Only those studies of acute community-acquired pneumonia underscored the utility of the measurement of blood pH, but in the context of severity scores combined with other parameters [68, 69].

#### Is lactate measurement useful to identify critically ill patients?

R2.2—Hyperlactatemia, whatever its value, should be considered as a marker of severity in initial treatment. Diagnostic and therapeutic management should be rapid and multidisciplinary if needed (GRADE 1+, STRONG AGREEMENT).

R2.3—Increase in blood lactate should probably be monitored in the first hours of management so as to assess the response to treatment (GRADE 2+, STRONG AGREEMENT).

*Rationale* Numerous studies show an association between initial blood lactate and the prognosis of septic shock and trauma. They are mostly retrospective cohort studies or prospective observational studies. Their methodologies are often questionable and their levels of proof limited. Nonetheless, all the studies agree on the utility of early measurement of arterial or venous lactate in evaluating the severity of septic shock and the need for critical care [70]. Hyperlactatemia is an independent index of severity and an initial lactate level above 4 mmol/L in septic shock [71] and above 2 mmol/L in trauma patients [72–74] is always associated with a worse prognosis [71, 75].

Several studies report the additional prognostic usefulness of plasma lactate decrease (clearance). The best cut-off seems to be 30% lactate clearance at the sixth hour of treatment in septic shock [76]. Likewise, no lactate decrease or a decrease of less than 20% in the first 2- to 4-h is associated with a worse prognosis in trauma patients [77].

Initial hyperlactatemia is also associated with a greater treatment burden. Pre-admission measurement of lactate improves identification of patients needing intensive care [43].

#### Does intensive monitoring of patients with diabetic ketoacidosis improve prognosis?

R2.4—The experts suggest close monitoring of patients with diabetic ketoacidosis, ideally in intensive care unit (EXPERT OPINION).

*Rationale* The indication for admission to intensive care is clear in the case of organ failure associated with ketoacidosis. However, for several decades certain studies have suggested that patients with uncomplicated diabetic ketoacidosis can be managed by conventional hospital care [78–80]. One retrospective cohort study in over 15,000 patients in 159 American hospitals showed that the use of intensive care for diabetic ketoacidosis patients was not associated with differences in mortality or length of hospital stay [81]. However, the results are difficult to interpret and generalize as it was a retrospective study based on coding data in which no clinical or paraclinical finding concerning the severity of ketoacidosis was provided. Moreover, the criteria of admission to intensive care were not indicated. As continuous intravenous insulin therapy is generally necessary and potentially serious complications can appear during therapeutic management (hypokalemia, hypoglycemia, pulmonary edema, cerebral edema), close clinical and paraclinical monitoring is indispensable. Since this monitoring may be compromised in conventional hospital care because of organizational difficulties, patients with diabetic ketoacidosis should be admitted to intensive care so as to adapt the treatment and watch for potential side effects.

### Third area: Therapeutic management

#### During diabetic ketoacidosis, what route of insulin delivery should be preferred?

R3.1—Insulin should probably be administered intravenously rather than subcutaneously in patients with diabetic ketoacidosis (GRADE 2+, STRONG AGREEMENT).

*Rationale* Two literature reviews have considered the optimal route for administration of insulin in diabetic ketoacidosis [82, 83]. Four controlled and randomized trials compared subcutaneous (SC) insulin with intravenous (IV) insulin in management of diabetic ketoacidosis in adults [84–87]. All evaluated the rate of correction of acidosis or the normalization of blood glucose. Three evaluated the length of hospital stay [85–87]. A lack of precision in the reported results meant that one of these studies [84] could not be included in the meta-analysis of the rate of correction of acidosis or normalization of blood glucose. This trial described a correction of ketosis and a significantly greater decrease in blood glucose at 2 h in the IV group, but non-significant results 4, 6, and 8 h after the start of therapeutic management. A meta-analysis of two trials comparing similar insulins [85, 87] found no significant difference in the rate of correction of acidosis or the normalization of blood glucose (difference = 0.2 h; 95% confidence interval [− 1.7–2.1]; *p* = 0.81). The last trial [86] reported similar results (*d* = − 1 h [− 3.2–1.2]; *p* = 0.36). The meta-analyses found no significant difference in the effect of the route of administration on the length of hospital stay. The literature data do not show that IV insulin therapy is preferable to SC insulin therapy, in terms of the rate of correction of acidosis, the normalization of blood glucose, or the length of hospital stay. However, few patients were included and they presented uncomplicated ketoacidosis. In addition, SC injections of insulin were performed regularly and the frequency of injections could be a source of discomfort or even pain.

As a venous route was often necessary, the continuous IV route seems preferable so as to facilitate restoration of water–electrolyte balance, avoid repeated SC injections, and reduce the risk of hypoglycemia, while ensuring better control of the insulin dose administered.

#### During diabetic ketoacidosis, should an insulin bolus be administered before starting continuous intravenous insulin therapy?

R3.2—An insulin bolus should probably not be administered before starting continuous intravenous insulin therapy in patients with diabetic ketoacidosis (GRADE 2−, STRONG AGREEMENT).

*Rationale* A literature review of the use of an initial insulin bolus before initiation of continuous intravenous insulin therapy identified just one randomized controlled trial [88] and one observational study [89]. In the latter, the normalization of blood glucose and the length of hospital stay did not differ significantly between the bolus and non-bolus groups (change in blood glucose 60.1 ± 38.2 vs 56.0 ± 45.4 mg/dL/h, respectively; *p* = 0.54; length of hospital stay 5.6 ± 5.3 vs 5.9 ± 6.9 days; *p* = 0.81). The authors noted more cases of hypoglycemia in the bolus group, but the difference was not statistically significant (6 vs 1%; *p* = 0.12). The randomized controlled trial compared three groups: a low-dose insulin bolus then a low insulin dose (0.07 IU/kg, then 0.07 IU/kg/h), a low insulin dose without an initial bolus (0.07 IU/kg/h), and double-dose insulin (0.14 IU/kg/h) without an initial bolus. The rate of correction of acidosis, the normalization of blood glucose, and the length of hospital stay did not differ between the three groups. It is important to note that this study did not evaluate the insulin dose commonly used in continuous intravenous administration, i.e., 0.1 IU/kg/h.

#### During diabetic ketoacidosis, should high or low continuous intravenous insulin doses be administered?

R3.3—Low continuous intravenous insulin doses should probably be administered in the treatment of diabetic ketoacidosis (GRADE 2+, STRONG AGREEMENT).

R3.4—The experts suggest using an initial dosage of 0.1 IU/kg/h without exceeding 10 IU/h, and to increase it in the absence of hypokalemia, if the targets for correction of blood ketones (0.5 mmol/L/h), bicarbonate (3 mmol/L/h), and capillary blood glucose (3 mmol/L/h) are not reached after the first hours of treatment (EXPERT OPINION).

*Rationale* The literature data, essentially from the 1970s, indicate that low continuous intravenous insulin doses are as effective as higher doses [90, 91]. A literature review found two trials (with no control group) reporting a decrease in blood glucose that was similar for low and high insulin doses. The risk of hypokalemia, hypoglycemia, or cerebral edema possibly associated with high doses and efficacy of low doses have justified their use in practice for several decades. However, if the targets for correction of blood ketones (0.5 mmol/L/h) or failing that of bicarbonate (3 mmol/L/h) and blood glucose (3 mmol/L/h) are not reached, it is possible to envisage increased doses, provided there is no hypokalemia.

#### Should sodium bicarbonate infusion be used in severe metabolic acidosis and, if so, in what situations?

R3.5—The experts suggest administering sodium bicarbonate to compensate for gastrointestinal or renal base loss in case of poor clinical tolerance (EXPERT OPINION).

*Rationale* The administration of sodium bicarbonate could limit the deleterious cardiovascular, respiratory, and cellular energy effects of loss of bicarbonate [2]. Sodium bicarbonate should be administered carefully as it is associated with a risk of hypokalemia, hypernatremia, hypocalcemia, rebound alkalemia, and water–sodium overload [2].

R3.6—Sodium bicarbonate should probably be administered to intensive care patients with severe metabolic acidemia (pH ≤ 7.20, PaCO_2_ < 45 mmHg) and moderate to severe acute renal insufficiency (GRADE 2+, STRONG AGREEMENT).

*Rationale* Metabolic acidosis accompanying states of shock is often multifactorial, with hyperlactatemia and renal insufficiency being involved first and foremost, plus potentially associated loss of bicarbonate. Several retrospective, observational, single-center [92–94] or prospective, multicenter studies [95] were insufficient to draw conclusions regarding the role of sodium bicarbonate. Two randomized, prospective, crossover, single-center physiological studies in 10 [96] and 14 patients [97] concluded that administration of sodium bicarbonate did not have a more favorable effect than saline solution on hemodynamic parameters measured by pulmonary arterial catheter in patients with metabolic lactic acidosis (blood bicarbonate ≤ 22 or 17 mmol/L and arterial blood lactate > 2.5 mmol/L).

A controlled, randomized, prospective multicenter study in 400 patients (pH ≤ 7.20, blood bicarbonate ≤ 20 mmol/L and PaCO_2_ ≤ 45 mmHg and blood lactate > 2 mmol/L or SOFA score > 4) compared the effect of sodium bicarbonate administration (4.2% q.s. pH ≥ 7.30) with the absence of such administration on a principal composite endpoint (day-28 mortality and/or presence of at least one organ failure at day 7, according to the SOFA score). The authors reported no effect of alkalinization (71% of patients in the control arm and 66% of patients in the bicarbonate arm reached the composite endpoint; the estimated absolute difference was − 5.5% ([95% CI − 15.2% to 4.2%], *p* = 0.24). The probability of day 28 survival was 46% [95% CI − 40% to 54%] in the control group and 55% [95% CI 49% to 63%]; *p* = 0.09 in the bicarbonate group.

In the a priori defined stratum “acute renal insufficiency—AKIN 2–3,” 74 (82%) of the 90 patients of the control group and 64 (70%) of the 92 patients of the bicarbonate group reached the composite endpoint (estimated absolute difference: − 12.3%, 95% CI − 26.0% to − 0.1%; *p* = 0.0462). The probability of survival at day 28 was 46% [95% CI 35% to 55%] in the control group and 63% [95% CI 52% to 72%] in the bicarbonate group (*p* = 0.0283).

These results were confirmed in multivariate analysis. In the general population and the “acute renal insufficiency” stratum, the patients randomized to the control arm received renal replacement therapy (RRT) more often and for longer than the patients of the bicarbonate arm (52% need for RRT in the control arm vs 35% in the bicarbonate arm, *p* < 0.001) [98].

R3.7—Sodium bicarbonate should not be administered routinely in the therapeutic management of circulatory arrest, apart from pre-existing hyperkalemia or poisoning by membrane stabilizers (GRADE 1−, STRONG AGREEMENT).

*Rationale* Since the 1999 French consensus conference, the role of sodium bicarbonate alkalinization in the therapeutic management of the cardiac arrest has been evaluated in 5 retrospective studies [99–103] and a prospective, randomized, double-blind, controlled multicenter study [104]. Four retrospective studies showed an increase in the frequency of resumption of spontaneous circulatory activity in patients treated with sodium bicarbonate [99, 101–103] and one reported decreased hospital survival in patients treated with sodium bicarbonate [100]. The randomized clinical trial (792 patients) found no difference in survival between the patients treated with sodium bicarbonate (7.4%) and those receiving a placebo (6.7%, *p* = 0.88). The use of sodium bicarbonate in patients could be reserved for pre-existing hyperkalemia or poisoning by membrane stabilizers [105].

R3.8—Sodium bicarbonate should probably not be administered to patients with diabetic ketoacidosis (GRADE 2−, STRONG AGREEMENT).

*Rationale* The administration of sodium bicarbonate transiently increases pH and may limit the deleterious cardiovascular and cellular energy effects of acidemia. However, the administration of sodium bicarbonate is associated with a risk of hypokalemia, hypernatremia, hypocalcemia, rebound alkalemia, and water–sodium overload [2]. A pathophysiological study in 39 patients has recently shown altered microvascular endothelial reactivity at the acute phase of diabetic ketoacidosis. This endothelial dysfunction was more marked when the arterial pH was below 7.20 and the vascular reactivity improved after 24 h of treatment. However, the administration of sodium bicarbonate was not tested in this observational study [106].

Since the 1999 French consensus conference, the role of sodium bicarbonate alkalinization in therapeutic management of ketoacidosis was reassessed in a retrospective single-center study [107] comparing 44 patients treated with bicarbonate and 42 untreated patients. The authors found no effect of sodium bicarbonate on the rate of correction of acidemia, as in previous studies, all of which were conducted in small populations [108].

R3.9—The experts suggest administering sodium bicarbonate in the therapeutic management of salicylate poisoning, whatever the pH value (EXPERT OPINION).

*Rationale* Salicylate poisoning is rare and potentially fatal. Toxicological expertise is needed to ensure optimal therapeutic management. The aim of bicarbonate administration is twofold: induce alkalemia to limit the passage of salicylate into the central nervous system and alkalinization of urine to promote renal excretion of salicylate [109, 110]. An old observational study in a small number of patients suggested that simple alkalinization leads to renal excretion of salicylate equal to or even greater than that of forced diuresis, alkaline diuresis, or not [111]. The administration of sodium bicarbonate should be subject to close monitoring as it is associated with a risk of hypokalemia, hypernatremia, hypocalcemia, alveolar hypoventilation, and fluid overload [2, 109]. In the case of severe poisoning, the experts suggest renal replacement therapy (cf. R3.13) and continued alkalinization between renal replacement therapy sessions until salicylate is completely eliminated.

#### Should renal replacement therapy be used in severe metabolic acidosis, and if so in what situations?

R3.10 In case of shock and/or acute renal insufficiency, the experts suggest initiation of renal replacement therapy if the pH is below or equal to 7.15 in the absence of severe respiratory acidosis and despite appropriate treatment (EXPERT OPINION).

*Rationale* There are no randomized controlled studies with mortality as the main endpoint that compare the initiation or not of renal replacement therapy in severe metabolic acidosis. The recommendations presented here come mostly from retrospective observational studies and case reports.

According to a questionnaire administered by the European Society of Intensive Care Medicine, 74% of intensivists consider metabolic acidosis (without indication of severity) to be a criterion for initiation of renal replacement therapy [112].

The plasma bicarbonate or pH cut-off authorizing renal replacement therapy could be deduced from the results of randomized studies comparing the effect on mortality of early or delayed initiation of renal replacement therapy in acute renal insufficiency. In 101 surgery patients, Wald et al. [113] found no difference in mortality as a function of the timing of renal replacement therapy, and plasma bicarbonate at its initiation was similar in the two groups: 20.7 ± 4.3 vs 20.1 ± 4.4 mmol/L. In 231 surgery patients with KDIGO stage-2 acute renal insufficiency, Zarbock et al. [114] found at initiation of renal replacement therapy similar plasma bicarbonate levels in the early and late arms: 20.9 ± 3.6 mmol/L vs 20.7 ± 3.7 mmol/L. Mortality was significantly lower in the early initiation group.

In the AKIKI study [115] in 619 patients with KDIGO stage-3 acute renal insufficiency, intention-to-treat analysis showed that pH and plasma bicarbonate were significantly lower in the late renal replacement therapy group (hard criteria for renal replacement therapy, including pH ≤ 7.15, rate of renal replacement therapy: 50%) than in the early renal replacement therapy group (6 h after inclusion, rate of renal replacement therapy: 100%): bicarbonate 16.6 ± 5.6 vs 18.9 ± 4.9 mmol/L (*p* < 0.001) and pH 7.25 ± 0.15 vs 7.30 ± 0.12 (*p* < 0.001). There was no difference in mortality between groups.

The IDEAL ICU study [116] included 488 septic shock patients with RIFLE stage F acute renal insufficiency randomized to 2 arms (renal replacement therapy started within 12 h following inclusion, rate of renal replacement therapy 97% versus renal replacement therapy started 48 h after inclusion in the absence of resolution of acute renal insufficiency, rate of renal replacement therapy: 62%). There was no difference in mortality (58 vs 54%) and the study was stopped as medical care was deemed futile. A pH ≤ 7.15 was a criterion for initiation of renal replacement therapy. Of the 41 patients in the late arm, 20 had a pH of 7.10.

The BICAR-ICU study [98] compared intravenous administration of 4.2% sodium bicarbonate (q.s. pH > 7.30) with a control arm without infusion of bicarbonate in patients with severe metabolic acidosis (pH ≤ 7.20, bicarbonate < 20 mmol/L and PaCO_2_ ≤ 45 mmHg) and a SOFA score ≥ 4 or arterial blood lactate ≥ 2 mmol/L. This randomized, controlled, intention-to-treat study was stratified according to age, AKIN stage 2 or 3 acute renal insufficiency, and septic shock. Renal replacement therapy was used if 2 of the 3 following criteria applied: pH < 7.20 after 24 h, hyperkalemia, or urine output < 0.3 mL/kg/h over 24 h. In the acute renal insufficiency sub-group of 182 patients, the probability of survival at day 28 was 46% [95% CI 35% to 55%] in the control group and 63% [95% CI 52% to 72%] in the bicarbonate group (*p* = 0.0283).

R3.11—In case of lactic acidosis suggestive of metformin poisoning, the experts suggest early initiation of renal replacement therapy when there is an organ dysfunction or in the absence of improvement in the first hours of therapeutic management (EXPERT OPINION).

*Rationale* Metformin-associated lactic acidosis is defined by arterial lactate above 5 mmol/L and pH below 7.35 during metformin treatment. Its incidence is low: from 10 to 12/100,000 [117, 118]. A 2015 literature review identified 175 publications (no randomized trial) reporting high mortality (30 to 50%) [119].

Yeh H-C et al. [117] collated case reports and studies from 1977 to 2014 (3 studies, 142 case reports) in 253 patients and found a mortality of 16.2%. Factors associated with mortality were mechanical ventilation and lactate level (17 vs 22 mmol/L, *p* < 0.01), but not pH, plasma bicarbonate, or level of metformin. A lactate level above 20 mmol/L was significantly associated with mortality.

A retrospective study conducted in Northern Italy from 2010 to 2015 collated 117 cases and reported 78.3% survival [118]. On average, at initiation of renal replacement therapy, the pH was below 7.04 and blood lactate above 12 mmol/L.

As the metformin dose is not always available and its prognostic value is subject to discussion [119], renal replacement therapy should be initiated without delay when there is an organ dysfunction or when there is no improvement in the first hours of therapeutic management. Renal replacement therapy is intended to correct water–electrolyte and acid–base disturbances and to ensure metformin clearance [119].

R3.12—In case of methanol or ethylene glycol poisoning, the experts suggest initiation of renal replacement therapy if the anion gap is above 20 mEq/L or if there is renal insufficiency or visual impairment (EXPERT OPINION).

*Rationale* Methanol poisoning and ethylene glycol poisoning are rare and potentially fatal. Expertise is needed to ensure optimal therapeutic management including, if necessary, specific intensive care procedures.

In alcohol poisoning (methanol and ethylene glycol), the pH at admission is correlated with the prognosis [120, 121]. A pH below 7.0 is predictive of death [122], whereas a pH above 7.22 is associated with survival [123]. The plasma anion gap (> 24 mEq/L or > 20 mEq/L in the case of hemodynamic instability) is correlated with the level of formate and with the prognosis [124].

Circulating methanol is removed by the kidney with a clearance of about 5 to 6 mL/min, which represents approximately 25 to 50% of its systemic elimination before its conversion to formic acid (responsible for the toxicity). This conversion is inhibited by intravenous administration of ethanol or fomepizole. The clearance of methanol achieved by intermittent hemodialysis ranges between 77 and 400 mL/min, and between 17 and 48 mL/min if renal replacement therapy is continuous [125, 126].

R3.13—In metabolic acidosis associated with salicylic acid poisoning, the experts suggest initiation of renal replacement therapy when there is neurological involvement and/or if the salicylic acid concentration is above 6.5 mmol/L (90 mg/dL) and/or if the pH is less than or equal to 7.20 (EXPERT OPINION).

*Rationale* Salicylate poisoning is rare and potentially fatal. Expertise is needed to ensure optimal therapeutic management comprising, if necessary, specific intensive care procedures.

A 2015 literature review by a group of experts [110] found 84 publications, 80 of which related to case reports or patient cohorts and to a randomized controlled trial, and collated 143 patients with salicylate poisoning. The authors concluded that salicylic acid is highly dialyzable and that intermittent hemodialysis is the preferred modality. They also concluded that development of acidemia should be considered as a warning sign because it indicates the onset of an organ dysfunction (lactic acidosis, ketoacidosis, renal, and/or respiratory insufficiency). In addition, the presence of acidemia increases the entry of salicylate into the central nervous system and the risk of cerebral edema.

A more recent retrospective study [127] in 56 mechanically ventilated patients with blood salicylate above 50 mg/dL reported 76% mortality. Failure to use renal replacement therapy was associated with increased mortality and survival was zero when blood salicylate was above 5.8 mmol/L, i.e., 80 mg/dL. However, no data were available on potential poisoning with other compounds or on the causes of death.

Given the limited volume and quality of the data, it is difficult to determine a toxic threshold accurately. However, it appears that above 6.5 mmol/L (90 mg/dL) the risk of death is high, even in the absence of clinical signs.

#### Should minute ventilation be increased in mechanically ventilated patients with metabolic acidosis?

R3.14—The experts suggest compensating for acidemia by increasing respiratory frequency without inducing intrinsic positive end-expiratory pressure, with a maximum of 35 cycles/min and/or a tidal volume up to 8 mL/kg of body mass, and by monitoring plateau pressure. The aim of ventilation is not to normalize pH. A target pH greater than or equal to 7.15 seems reasonable. Medical treatment of metabolic acidosis and of its cause should be envisaged concomitantly, as ventilatory compensation can only be symptomatic and temporary (EXPERT OPINION).

*Rationale* The control of breathing brings into play three types of interconnected structures: the control center commonly called the “respiratory centers” in the central nervous system at the level of the brainstem; the motor components of the respiratory system comprising the muscles of the upper airways, the thoracic cage, and the abdomen; and the receptors (chemoreceptors, muscle proprioceptors, airway and lung receptors) that transmit setpoints constantly to the respiratory centers (PCO_2_, pH, lung distension, respiratory muscle load…). Thus, the respiratory centers receive sensory and humoral information that enables homeostasis, while optimizing the energy cost of each respiratory cycle. The central chemoreceptors on the ventral side of the brainstem respond rapidly and strongly to minimal variations in the pH and PCO_2_ of the cerebrospinal fluid and blood. The hydrogen ion seems to be a determinant stimulus [128].

In metabolic acidosis, the physiological response is an increase in alveolar ventilation [129] that is constant, whatever the cause and severity of acidosis [130]. The stimulation of chemoreceptors in metabolic acidosis is responsible for an increase in tidal volume rather than tachypnea [130, 131]. Its efficacy depends not only on alveolar ventilation, but also on the hemodynamic state and integrity of the respiratory system [129, 132].

As yet there are no specific data concerning ventilatory management of intubated-ventilated patients with metabolic acidosis. Though acidosis is conventionally associated with a poor prognosis [133], it has potentially protective effects. Apart from the severity of acidosis, its mechanism and how it arises seem to be prognostic factors that should be taken into account.

The correction of metabolic acidosis by increasing respiratory frequency and/or tidal volume is questionable. Current data on protective ventilation are abundant and recommend keeping a tidal volume of about 6 mL/kg of body mass. Given the hemodynamic effects of metabolic acidosis, it seems reasonable to adapt the respiratory frequency to achieve a pH greater than or equal to 7.15 [134–136], without exceeding 35 cycles/min, as data in animal models suggest that a high minute ventilation has deleterious effects [137, 138], which are more marked when there is lung involvement.

## Abbreviations

BE: base excess
SBE: standard base excess
AG: anion gap
cAG: corrected anion gap
SID: strong ion difference
eff SID: effective strong ion difference
app SID: apparent strong ion difference
IV: intravenous
SC: subcutaneous
RRT: renal replacement therapy
